# Molecular Epidemiology of St. Louis Encephalitis Virus, São Paulo State, Brazil, 2016–2018

**DOI:** 10.3201/eid3105.250158

**Published:** 2025-05

**Authors:** Giovana Santos Caleiro, Ingra M. Claro, Xinyi Hua, Giovana V. Basile, Karolina M.B. Nuevo, Charlys A. da Costa, Rosa M. Tubaki, Regiane M.T. de Menezes, Sirle A.S. Scandar, Lilian A.R. Colebrusco, Emerson L.L. Araújo, William M. de Souza, Ester C. Sabino, Nuno R. Faria, Mariana Sequetin Cunha

**Affiliations:** University of São Paulo, São Paulo, Brazil (G.S. Caleiro, I.M. Claro, C.A. da Costa, E.C. Sabino, N.R. Faria); Instituto Adolfo Lutz, São Paulo (G.S. Caleiro, G.V. Basile, K.M.B. Nuevo, M.S. Cunha); University of Kentucky, Lexington, Kentucky, USA (I.M. Claro, X. Hua, W.M. de Souza); Pasteur Institute, São Paulo, São Paulo (R.M. Tubaki, R.M.T. de Menezes, S.A.S. Scandar, L.A.R. Colebrusco); Ministry of Health, Brasília, Brazil (E.L.L. Araújo); Imperial College London, London, UK (N.R. Faria)

**Keywords:** St. Louis encephalitis virus, SLEV, viruses, meningitis/encephalitis, zoonoses, mosquitoes, vector-borne infections, Aedes, Sabethes, Brazil

## Abstract

We detected St. Louis encephalitis virus (SLEV) in 0.16% (3/3,375) of *Aedes* and *Sabethes* spp. mosquitoes captured during 2016–2018 in São Paulo State, Brazil. We also isolated and confirmed that the SLEV strains belong to genotype III. Continued surveillance is required to clarify the burden of SLEV in Brazil.

St. Louis encephalitis virus (SLEV; species *Orthoflavivirus louisense*) is an endemic mosquitoborne orthoflavivirus in the Americas that can cause febrile and central nervous system diseases in humans, neurologic illnesses in equids, and fatal outcomes in both (*1*,*2*). SLEV is transmitted in enzootic and epizootic transmission cycles mainly by *Culex* and related genera of mosquitoes; humans and equids are incidental and dead-end hosts (*1*). In South America, sporadic SLEV human cases have been reported since 1953, and numerous serosurveys indicated SLEV circulation (*1*,*3**–**5*). However, reports of active SLEV circulation remain scarce in South America. Here, we conducted a molecular epidemiology study in mosquitoes collected during 2016–2018 in São Paulo state, Brazil.

During November 2016–June 2019, we captured Culicinae mosquitoes across 9 genera and combined the samples into 3,375 pools for analysis. The most abundant species were *Aedes scapularis* (26.5%, 893/3,375) and *Ae. albopictus* (21.7%, 731/3,375) (Appendix). Next, we extracted RNA from all homogenized mosquito pools and performed real-time reverse transcription PCR (RT-PCR) to detect flavivirus RNA (*6*). We detected flavivirus RNA in 0.16% (3/3,375) of the mosquito pools. Positive mosquito pools were *Ae. albopictus* (strain no. MO239, n = 3 specimens), *Ae. aegypti* (strain no. MO1424, n = 1 specimen), and *Sabethes chloropterus* (strain no. MO730, n = 1 specimen), which were collected during November 24, 2016–February 16, 2017, in São José do Rio Preto and Araçatuba municipalities (Appendix). Subsequently, we conducted viral isolation in *Ae. albopictus* clone C6/36 cells from positive samples. We isolated all 3 positive strains in C6/36 cells and confirmed by immunofluorescent staining and real-time RT-PCR. Then, we used nanopore sequencing (*7*) to obtain nearly complete coding sequences (>99%) for 6 SLEV strains, at an average coverage >20-fold/nucleotide. We submitted all sequences to GenBank (accession nos. PP855630–4 and PP871388). 

To contextualize SLEV circulation in São Paulo state, we sequenced 3 SLEV isolates identified in São Paulo state during 1993–2004 that had been either partially sequenced or never sequenced (Appendix). Next, we conducted a maximum-likelihood phylogenetic analysis that showed that the SLEV strains MO239, MO1424, and MO730 from this study, and historical strain SPH253157 clustered in a well-supported clade (100% bootstrap) at the basal of genotype III (Figure). In addition, historical SLEV strains SPAR149623 and SPAR147631 clustered within genotype V, along with strains identified in Brazil and Peru during 1973–2006 and in the United States in 2003 (Figure). The SLEV strains we sequenced shared 98.6%–98.9% nucleotide identity with other genotype III strains and 99.8%–99.9% nucleotide identity among themselves. The newly sequenced genotype V strains showed a 93.3% nucleotide divergence from the genotype III strains. We identified 75 aa substitutions across the sequenced SLEV strains; most substitutions were in the envelope and nonstructural 4B proteins (16.0%, 12/75 in each) and the nonstructural 5 protein (38.7%, 29/75) (Appendix).

**Figure:**
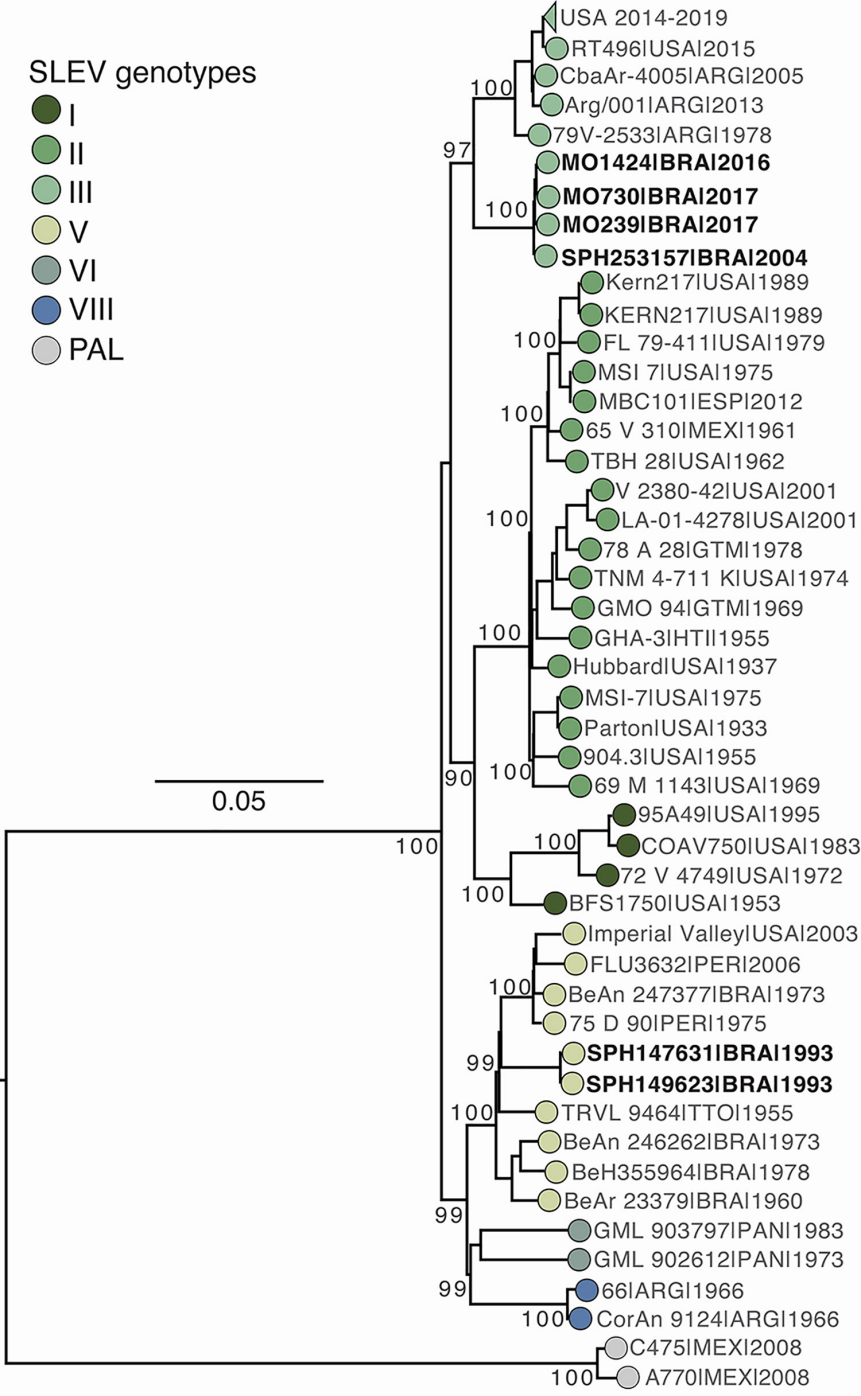
Maximum-likelihood phylogenetic tree in a study of molecular epidemiology of St. Louis encephalitis virus in São Paulo State, Brazil, 2016–2018. Tree shows 219 representative SLEV complete coding sequence genomes, including 3 new genomes generated in this study from *Aedes albopictus* (MO239), *Ae. aegypti* (MO1424), and *Sabethes chloropterus* (MO730) mosquitoes. The tree also includes 3 historical partially sequenced or unsequenced SLEV isolates from São Paulo state: SPAR149623 (*Culex* sp. mosquito, Santo Antônio de Aracanguá, May 12, 1993), SPAR147631 (*Anopheles triannulatus* mosquito, Pereira Barreto, March 11, 1993), and SPH253157 (human, São Pedro, January 1, 2004). Tree tips are colored by genotype. Phylogenies are midpoint-rooted for clarity; bootstrap values (1,000 replicates) are shown on major nodes. Scale bar represents nucleotide substitutions per site. GenBank accession numbers for the sequences and detailed information on the collapsed USA clade (genotype III, circulating during 2014–2019) is provided in the Appendix. SLEV, St. Louis encephalitis virus.

This study reports the active circulation of SLEV during 2016–2018 in São Paulo state, Brazil. We found that SLEV genotype III continues to circulate in Brazil in *Aedes* and *Sabethes* spp. mosquitoes since the human case that was reported in 2004 (*8*). SLEV genotype III has caused sporadic outbreaks in Argentina since 1979 (*6*). In 2014, SLEV genotype III was identified in the western United States, presumably introduced from South America by migratory birds (*9*). We detected SLEV in *Ae. albopictus*, *Ae. aegypti*, and *Sa. chloropterus* mosquitoes, but the role of those mosquito species in SLEV transmission requires further field and experimental entomological studies. Also, the *Sa. chloropterus* mosquito is a vector of yellow fever virus in Brazil, but its role in SLEV transmission remains to be determined. 

The first limitation of our study is that we focused on the molecular detection of SLEV in mosquitoes, which provides insight into active infections, but further investigations are required to assess SLEV circulation in vertebrates, including humans, through molecular and serologic methods. Serologic methods in areas with cocirculating flaviviruses present challenges because of potential cross-reactivity (*10*); however, this approach can substantially contribute to our understanding of the extent of both current and past SLEV infections in the region. Second, our mosquito sampling was predominately *Aedes* spp., but because *Culex* spp. mosquitoes are the primary vector for SLEV, more research is needed to determine the dynamic transmission of SLEV in Brazil. Finally, the lack of blood meal analysis in our study prevents the identification of potential amplifying hosts for SLEV in the region and the determining vector feeding preferences.

In conclusion, our study demonstrates active SLEV circulation in mosquitoes in São Paulo state, Brazil. These findings emphasize the need for continued surveillance efforts using a One Health concept to understand the transmission dynamics and ecologic drivers of SLEV.

Appendix Additional information for molecular epidemiology of St. Louis encephalitis virus, São Paulo state, Brazil, 2016–2018.
