# Supplementary material for: Molecular Epidemiology of St. Louis Encephalitis Virus, São Paulo State, Brazil, 2016–2018
Source: Emerg Infect Dis. 2025 May;31(5):1052–4. doi: 10.3201/eid3105.250158 (PMC12044246; doi:10.3201/eid3105.250158)
Supplement: Appendix — Additional information for molecular epidemiology of St. Louis encephalitis virus, São Paulo state, Brazil, 2016–2018. [file 25-0158-Techapp-s1.pdf]

Article DOI: https://doi.org/10.3201/eid3105.250158

EID cannot ensure accessibility for supplementary materials supplied by authors. Readers who have difficulty accessing supplementary content should contact the authors for assistance.

Molecular Epidemiology of St. Louis Encephalitis Virus, São Paulo State, Brazil, 2016–2018

Appendix

Mosquitoes sampling and pool preparation

We conducted entomological surveillance between November 2016 and June 2019 in urban and periurban areas of 29.8% (192/645) of municipalities in São Paulo State, Brazil (Appendix Figure 1). Mosquitoes were collected at ground level from dawn to dusk using an entomologic net and bottle-type manual vacuums in green areas, while Nasci aspirator was used in urban dwellings. Then, mosquitoes were classified based on morphological identification, and females were placed into pools containing between 1 and 50 mosquitoes according to species, date, and location of collection. Next, mosquito pools were homogenized in a Magna Lyser (Roche, Switzerland) using 0.6 mL of phosphate-buffered saline (PBS) containing 0.75% bovine albumin, 100 units/mL penicillin, and 100 µg/mL streptomycin. Then, homogenate was centrifuged at 1800 × g for 15 minutes. The supernatant was collected and stored at -80°C for subsequent analysis

RNA Extraction and SLEV Detection

Viral RNA was extracted from mosquito pools using the QIAamp Viral RNA Mini Kit according to the manufacturer's instructions (Qiagen, Germany). Then, extracted RNA was subjected to RT-PCR for *Flavivirus* genus detection (*1*). Positive samples were subjected to viral isolation in C6/36 cells. Briefly, 20 µL of each positive pool was inoculated onto monolayers of C6/36 cells in cell culture tubes containing 10% fetal bovine serum (FBS). After a 1-hour adsorption period, the inoculum was removed, and the cells were incubated for nine days at 28°C

in L-15 medium supplemented with 2% FBS, 100 units/mL penicillin, and 100 μg/mL streptomycin. Three passages were performed. Viral isolation was confirmed by indirect immunofluorescence assay using a pool of in-house flavivirus hyperimmune polyclonal antibodies and a fluorescein isothiocyanate-labeled anti-mouse IgG (whole molecule) antibody (Sigma-Aldrich, USA). In-house polyclonal antibodies against flaviviruses are serum pooled from mice immunized with SLEV, as well as dengue, yellow fever, Zika, and Rocio viruses.

St Louis Encephalitis strains

We also performed genome sequencing of three historical SLEV strains previously unsequenced or partially sequenced: SPAR149623, isolated from *Culex* spp. mosquitoes in Santo Antônio de Aracanguá municipality in May 1993 (*1*); SPAR147631, isolated from *Anopheles triannulatus* in Pereira Barreto municipality in May 1993; and SPH253157, obtained from a human case in January 2004 in São Pedro, which was previously partially sequenced (*2*).

Genome Sequencing and Analysis

RNA extracted from viral isolates (MO239, MO1424, SPH253157, SPAR147631, and PAR149623) was treated with TURBO DNase (Thermo Fisher Scientific, USA) to remove residual DNA and then concentrated using the Zymo RNA Clean & Concentrator-5 kit (Zymo Research, USA). cDNA synthesis and untargeted amplification were performed using the SMART-9N protocol (*3*). For Nanopore sequencing, MinION libraries were prepared using the EXP-NBD104 (1-12) and EXP-NBD114 (13-24) Native Barcoding Kits (Oxford Nanopore Technologies, UK). Libraries were sequenced on a MinION device (Oxford Nanopore Technologies, UK) using the SQK-LSK109 Kit and the FLO-MIN106 flow cells. Raw reads were demultiplexed using Guppy (Oxford Nanopore Technologies, UK) and taxonomically classified using the standard Kraken2 database (*4*). Identified SLEV reads were mapped to the SLEV reference genome (GenBank accession number NC_007580.2) using minimap2 version 2.22-r1101 (*5*). Consensus sequences were generated using Medaka (Oxford Nanopore Technologies, UK) for genome regions with at least 20-fold read depth coverage. SLEV strain MO730 was sequenced using the Illumina Miseq platform, as previously described (*6*).

Phylogenetic Analysis and Nucleotide Distance

Sequences generated in this study were aligned with complete coding sequences of SLEV strains available in the NCBI Virus Database (https://www.ncbi.nlm.nih.gov/labs/virus/vssi/#/) as of August 21, 2024. Multiple sequence alignments (MSA) were performed using MAFFT version 7.525 (https://mafft.cbrc.jp/alignment/software) as previously described (*7*) and manually adjusted in Aliview. Recombination events were screened using all available methods in RDP version 5 (*8*). Phylogenetic analysis was conducted using the GTR+I+G nucleotide substitution model, selected via ModelFinder in IQ-TREE, with 1,000 ultrafast bootstrap replicates to determine statistical support for the maximum-likelihood (ML) tree nodes (*8*,*9*). The resulting phylogenies were annotated and visualized using Figtree version 1.4.4 (http://tree.bio.ed.ac.uk/software/figtree). Pairwise nucleotide distances between sequences generated in the final dataset were calculated using Geneious Prime 2023.0.4 (https://www.geneious.com).

References

1. Cunha MS, Luchs A, Dos Santos FCP, Caleiro GS, Nogueira ML, Maiorka PC. Applying a pan-flavivirus RT-qPCR assay in Brazilian public health surveillance. Arch Virol. 2020;165:1863–8. PubMed https://doi.org/10.1007/s00705-020-04680-w

2. Santos CL, Sallum MAM, Franco HM, Oshiro FM, Rocco IM. Genetic characterization of St. Louis encephalitis virus isolated from human in São Paulo, Brazil. Mem Inst Oswaldo Cruz. 2006;101:57–63. PubMed https://doi.org/10.1590/S0074-02762006000100011

3. Claro IM, Ramundo MS, Coletti TM, da Silva CAM, Valenca IN, Candido DS, et al. Rapid viral metagenomics using SMART-9N amplification and nanopore sequencing. Wellcome Open Res. 2023;6:241. PubMed https://doi.org/10.12688/wellcomeopenres.17170.2

4. Wood DE, Lu J, Langmead B. Improved metagenomic analysis with Kraken 2. Genome Biol. 2019;20:257. PubMed https://doi.org/10.1186/s13059-019-1891-0

5. Li H. Minimap2: pairwise alignment for nucleotide sequences. Bioinformatics. 2018;34:3094–100. PubMed https://doi.org/10.1093/bioinformatics/bty191

6. Ramos EDSF, Rosa UA, Ribeiro GO, Villanova F, Milagres FAP, Brustulin R, et al. Multiple clades of Husavirus in South America revealed by next generation sequencing. PLoS One. 2021;16:e0248486. PubMed https://doi.org/10.1371/journal.pone.0248486

7. Katoh K, Misawa K, Kuma K, Miyata T. MAFFT: a novel method for rapid multiple sequence alignment based on fast Fourier transform. Nucleic Acids Res. 2002;30:3059–66. PubMed https://doi.org/10.1093/nar/gkf436

8. Martin DP, Varsani A, Roumagnac P, Botha G, Maslamoney S, Schwab T, et al. RDP5: a computer program for analyzing recombination in, and removing signals of recombination from, nucleotide sequence datasets. Virus Evol. 2020;7:veaa087. PubMed https://doi.org/10.1093/ve/veaa087

9. Minh BQ, Schmidt HA, Chernomor O, Schrempf D, Woodhams MD, von Haeseler A, et al. IQ-TREE 2: New Models and Efficient Methods for Phylogenetic Inference in the Genomic Era. Mol Biol Evol. 2020;37:1530–4. PubMed https://doi.org/10.1093/molbev/msaa015

Appendix Table 1. Information of SLEV isolates sequenced in this study.

Sample	Host	Collection Date	Municipality	Genotype	Accession Number
MO239	*Aedes albopictus*	2017-Jan-24	São José do Rio Preto	III	PP855630
MO1424	*Aedes aegypti*	2016-Nov-25	Araçatuba	III	PP855631
MO730	*Sabethes chloropterus*	2017-Feb-16	São José do Rio Preto	III	PP871388
SPAR149623	Culex spp.	1993-May-12	Santo Antônio de Aracanguá	V	PP855633
SPAR147631	*Anopheles triannulatus*	1993-Mar-11	Pereira Barreto	V	PP855634
SPH253157	Human	2004-Jan-01	São Pedro	III	PP855632

Appendix Table 2. Amino acid differences in the polyprotein gene among SLEV strains sequenced in this study, using the SPH253157 strain as the reference*

Proteins	SLEV sequence	Genome position	SLEV strain SPH253157	SLEV new strains
anchC	SPAR147631	120	S	N
anchC	SPAR149623	120	S	N
E	MO1424	444	F	S
E	MO239	444	F	S
E	MO730	444	F	S
E	SPAR147631	411	N	S
E	SPAR147631	439	S	T
E	SPAR147631	474	V	I
E	SPAR147631	604	T	A
E	SPAR147631	661	I	V
E	SPAR149623	411	N	S
E	SPAR149623	439	S	T
E	SPAR149623	604	T	A
E	SPAR149623	661	I	V
NS1	SPAR147631	862	K	R
NS1	SPAR147631	891	R	Q
NS1	SPAR147631	997	G	E
NS1	SPAR147631	1058	S	R
NS1	SPAR147631	1113	T	I
NS1	SPAR149623	862	K	R
NS1	SPAR149623	891	R	Q
NS1	SPAR149623	997	G	E

Proteins	SLEV sequence	Genome position	SLEV strain SPH253157	SLEV new strains
NS1	SPAR149623	1058	S	R
NS1	SPAR149623	1113	T	I
NS2A	SPAR147631	1150	L	F
NS2A	SPAR147631	1239	Q	K
NS2A	SPAR149623	1150	L	F
NS2A	SPAR149623	1239	Q	K
NS2B	SPAR147631	1416	R	K
NS2B	SPAR149623	1416	R	K
NS3	MO730	1880	K	R
NS3	MO239	1880	K	R
NS3	MO239	2054	Y	H
NS3	SPAR147631	1853	N	S
NS3	SPAR147631	2093	K	R
NS3	SPAR149623	1853	N	S
NS3	SPAR149623	2093	K	R
NS4B	MO239	2290	M	V
NS4B	MO239	2294	V	A
NS4B	SPAR147631	2280	A	T
NS4B	SPAR147631	2289	A	S
NS4B	SPAR147631	2290	M	V
NS4B	SPAR147631	2294	V	A
NS4B	SPAR147631	2297	T	I
NS4B	SPAR147631	2396	I	V
NS4B	SPAR147631	2514	I	V
NS4B	SPAR149623	2280	A	T
NS4B	SPAR149623	2289	A	S
NS4B	SPAR149623	2290	M	V
NS4B	SPAR149623	2294	V	A
NS4B	SPAR149623	2297	T	I
NS4B	SPAR149623	2396	I	V
NS4B	SPAR149623	2514	I	V
NS5 (RdRp)	SPAR147631	2578	Y	H
NS5 (RdRp)	SPAR147631	2664	S	P
NS5 (RdRp)	SPAR147631	2748	S	G
NS5 (RdRp)	SPAR147631	2759	S	N
NS5 (RdRp)	SPAR147631	2796	A	T
NS5 (RdRp)	SPAR147631	2954	E	G
NS5 (RdRp)	SPAR147631	2967	K	E
NS5 (RdRp)	SPAR147631	3031	Y	H
NS5 (RdRp)	SPAR147631	3090	R	K
NS5 (RdRp)	SPAR147631	3200	L	I
NS5 (RdRp)	SPAR147631	3361	V	A
NS5 (RdRp)	SPAR149623	2578	Y	H
NS5 (RdRp)	SPAR149623	2664	S	P
NS5 (RdRp)	SPAR149623	2748	S	G
NS5 (RdRp)	SPAR149623	2759	S	N
NS5 (RdRp)	SPAR149623	2796	A	T
NS5 (RdRp)	SPAR149623	2954	E	G
NS5 (RdRp)	SPAR149623	2967	K	E
NS5 (RdRp)	SPAR149623	3031	Y	H
NS5 (RdRp)	SPAR149623	3090	R	K
NS5 (RdRp)	SPAR149623	3200	L	I
NS5 (RdRp)	SPAR149623	3361	V	A

*SLEV, St. Louis encephalitis virus. anchC, anchored capsid protein. E, envelope protein. NS, nonstructural protein. RdRP, RNA-dependent RNA polymerase.

Appendix Table 3. Amino acid and nucleotide similarities of SLEV strains sequenced in this study, using the SPH253157 strain as the reference*

SLEV sequence	Amino acid similarity (%)	Nucleotide similarity (%)
MO1424	99.97	99.96
MO239	99.91	99.84
MO730	99.82	99.84
SPAR147631	98.89	93.30
SPAR149623	98.95	93.35

*SLEV, St. Louis encephalitis virus.

Appendix Table 4. Genome sequences used in the phylogenetic analyses.

GenBank ID	Strain	Host	Location	Collection date
JQ957868	Palenque-C475	Culex nigripalpus	Mexico	2008
JQ957869	Palenque-A770	Culex nigripalpus	Mexico	2008
KF589299	FLU3632	Human	Peru	2006-03-27
JF460774	Imperial Valley	Culex tarsalis	Imperial Valley, CA, USA	2003
EF158067	BeAn 247377	Hylophilax poecilonota	Para, Brazil	1973
EF158054	75 D 90	UNK	Peru	1975
EF158056	TRVL 9464	Psorophora ferox	Trinidad, Trinidad and Tobago	1955
KM267635	BeH355964	Human	Belem, PA, Brazil	1978
EF158053	BeAn 246262	Didelphis marsupialis	Para, Brazil	1973
EF158048	BeAr 23379	Sabethes belisarioi	Para, Brazil	1960
EF158060	GML 903797	UNK	Panama	1983
EF158064	GML 902612	Haemagogus equinus	Panama	1973
EF158063	CorAn 9124	Colomys musculinus	Cordoba, Argentina	1966
AY632544	Argentine 66	Colomys musculinus	Cordoba, Argentina	1966
EF158058	Kern 217	Culex tarsalis	Kern County, CA, USA	1989
MN233334	KERN217	Culex tarsalis	Kern County, CA, USA	1989
EF158062	FL 79-411	Culex nigripalpus	Florida, USA	1979
PP054842	MBC101	UNK	Spain	2012-03-22
EF158050	MSI 7	Passer comesticus	Mississippi, USA	1975
EF158059	65 V 310	UNK	Mexico	1961
EF158055	TBH 28	Human	Florida, USA	1962
MN233331	LA-01-4278	Culex quinquefasciatus	Ouachita Parish, LA, USA	2001-08-30
EF158052	V 2380-42	Culex quinquefasciatus	Texas, USA	2001
EF158057	78 A 28	UNK	Guatemala	1978
EF158065	TNM 4-711 K	Culex pipiens	Tennessee, USA	1974
EF158051	GMO 94	Culex nigripalpus	Guatemala	1969
EF158066	GHA-3	Butorides virescens	Haiti	1955
EU566860	Hubbard	Human	Missouri, USA	1937
EF158049	904.3	Colaptes auratus	Kentucky, USA	1955
EF158070	Parton	Human	Missouri, USA	1933
DQ359217	MSI-7	UNK	Mississippi, USA	1975
EF158061	69 M 1143	Procyon lotor (Mammal)	Florida, USA	1969
MN233332	BFS1750	Culex tarsalis	Kern County, CA, USA	1953
MN233333	COAV750	Culex tarsalis	Coachella Valley, CA, USA	1983
MH899073	95A49	Culicidae	Arizona, USA	1995
EF158069	72 V 4749	Culex tarsalis	Colorado, USA	1972
MW074966	3488	Culex tarsalis	USA	2017-09-06
MW074968	3490	Culex tarsalis	USA	2017-09-06
MW074969	3493	Culex tarsalis	USA	2017-09-06
MW074974	3531	Culex tarsalis	USA	2017-09-08
MW074978	3536	Culex tarsalis	USA	2017-09-08
MW074971	3504	Culex tarsalis	USA	2017-09-06
MW074970	3503	Culex tarsalis	USA	2017-09-06
MW074972	3513	Culex tarsalis	USA	2017-09-06
MW074975	3532	Culex tarsalis	USA	2017-09-08
MW074973	3526	Culex tarsalis	USA	2017-09-06
MW075029	pqMPlZov	Culex quinquefasciatus	USA	2017-08-30
MW074977	3535	Culex tarsalis	USA	2017-09-08
MN233308	COAV3064	Culex tarsalis	Coachella Valley, CA, USA	2017-07-26
MW074967	3489	Culex tarsalis	USA	2017-09-06
MW074976	3534	Culex tarsalis	USA	2017-09-08
MW074980	3620	Culex tarsalis	USA	2017-09-12
MW074979	3615	Culex tarsalis	USA	2017-09-12
MW074990	4455	Culex tarsalis	USA	2018-10-10
MW074982	2687	Culex tarsalis	USA	2018-07-03
MW074987	3581	Culex tarsalis	USA	2018-08-28
MW074991	3324	Culex tarsalis	USA	2018-08-14

GenBank ID	Strain	Host	Location	Collection date
MW074988	4073	Culex tarsalis	USA	2018-09-25
MW074994	3690	Culex tarsalis	USA	2018-09-05
MW074989	4421	Culex tarsalis	USA	2018-10-10
MW075095	nQ8K6oNM	Culex tarsalis	USA	2019-06-18
MW075096	dV7HaKQa	Culex tarsalis	USA	2019-06-24
MW075098	Y3tlog3B	Culex tarsalis	USA	2019-07-15
MW075100	v85x07rX	Culex stigmatosoma	USA	2019-07-11
MW075107	EMnoU6vq	Culex quinquefasciatus	USA	2019-07-25
MW075099	jcFWtjL9	Culex tarsalis	USA	2019-07-11
MW075106	O7uI3ibn	Culex quinquefasciatus	USA	2019-07-25
MW075101	FXC2p73C	Culex quinquefasciatus	USA	2019-07-17
MW075102	3DL0PtRd	Culex quinquefasciatus	USA	2019-07-17
MW074983	2900	Culex tarsalis	USA	2018-07-17
MW074985	3147	Culex tarsalis	USA	2018-07-26
MW074984	3000	Culex tarsalis	USA	2018-07-20
MW074986	3158	Culex tarsalis	USA	2018-07-31
MW074992	3404	Culex tarsalis	USA	2018-08-14
MW074993	3472	Culex tarsalis	USA	2018-08-21
MW074981	3622	Culex tarsalis	USA	2017-09-12
MW075049	5730	Culex quinquefasciatus	USA	2018-07-06
MW075050	6206	Culex tarsalis	USA	2018-06-21
MW075051	6162	Culex tarsalis	USA	2018-06-21
MW075092	8E88	Culex quinquefasciatus	USA	2019-08-06
MW075053	5760	Culex tarsalis	USA	2018-07-06
MW075052	5652	Culex tarsalis	USA	2018-07-06
MW075065	X652	Culex quinquefasciatus	USA	2019-05-08
MW075086	6D44	Culex quinquefasciatus	USA	2019-07-12
MW075034	J1IHzDWm	Culex quinquefasciatus	USA	2017-08-31
MW075082	3D31	Culex quinquefasciatus	USA	2019-07-02
MW075021	VdoK74JJ	Culex quinquefasciatus	USA	2017-08-16
MW075028	uUroCXhj	Culex tarsalis	USA	2017-08-30
MW075040	ia05Pxiv	Culex quinquefasciatus	USA	2017-09-17
MW075043	u0wHxDfi	Culex quinquefasciatus	USA	2017-10-31
MW074999	5cM80vCI	Culex quinquefasciatus	USA	2015-06-16
MW075032	bFd9N7qw	Culex quinquefasciatus	USA	2017-08-31
MW075030	kJ5aG818	Culex quinquefasciatus	USA	2017-08-31
MW075044	C001	Culex quinquefasciatus	USA	2018-10-23
MW075063	X575	Culex tarsalis	USA	2019-05-07
MW075069	X773	Culex quinquefasciatus	USA	2019-05-09
MW075075	Z162	Culex quinquefasciatus	USA	2019-05-15
MW075088	0E14	Culex tarsalis	USA	2019-07-17
MW075093	8E47	Culex quinquefasciatus	USA	2019-08-06
MW075089	6D56	Culex quinquefasciatus	USA	2019-07-17
MW075066	X688	Culex quinquefasciatus	USA	2019-05-08
MW075070	Z137	Culex quinquefasciatus	USA	2019-05-14
MW075090	8D58	Culex quinquefasciatus	USA	2019-07-24
MW075083	0E97	Culex tarsalis	USA	2019-07-03
MW075073	9A72	Culex tarsalis	USA	2019-05-15
MW075084	0E11	Culex tarsalis	USA	2019-07-10
MW075062	2A55	Culex tarsalis	USA	2019-05-07
MW075057	Z418	Culex quinquefasciatus	USA	2019-07-23
MW075061	X570	Culex tarsalis	USA	2019-05-01
MW075087	6D31	Culex tarsalis	USA	2019-07-17
MW075081	3D00	Culex tarsalis	USA	2019-07-02
MW075105	gknCtuxr	Culex quinquefasciatus	USA	2019-07-24
MW075058	3A34	Culex tarsalis	USA	2019-07-25
MW075064	X871	Culex tarsalis	USA	2019-05-07
MW075059	Z861	Culex quinquefasciatus	USA	2019-07-25
MW075072	Z132	Culex tarsalis	USA	2019-05-15
MW075103	iNdHUy8y	Culex quinquefasciatus	USA	2019-07-22
MW075104	fxf3ZrLO	Culex quinquefasciatus	USA	2019-07-23
MW075080	9A09	Culex quinquefasciatus	USA	2019-05-16
MW075071	X730	Culex quinquefasciatus	USA	2019-05-14
MW075056	2A84	Culex tarsalis	USA	2019-07-23
MW075078	9A07	Culex quinquefasciatus	USA	2019-05-16

GenBank ID	Strain	Host	Location	Collection date
MW075077	X399	Culex quinquefasciatus	USA	2019-05-15
MW075054	5356	Culex tarsalis	USA	2018-07-24
MW075036	tOGHz32t	Culex tarsalis	USA	2017-09-14
MW075025	gDLIPilu	Culex quinquefasciatus	USA	2017-08-23
MW075031	Cl5JARkA	Culex tarsalis	USA	2017-08-31
MW075022	ZmbJci8M	Culex quinquefasciatus	USA	2017-08-22
MW075076	X751	Culex quinquefasciatus	USA	2019-05-15
MW075091	5D98	Culex tarsalis	USA	2019-07-25
MW075094	Zw9wIdAm	Culex tarsalis	USA	2019-06-18
MW075097	cEdbDSdI	Culex tarsalis	USA	2019-07-01
MW075038	1OdNIUII	Culex quinquefasciatus	USA	2017-09-21
MW075055	4272	Culex tarsalis	USA	2018-07-25
MW075067	3A73	Culex quinquefasciatus	USA	2019-05-08
MW075041	1ATouSYI	Culex quinquefasciatus	USA	2017-10-13
MW075026	ZuleYNYt	Culex quinquefasciatus	USA	2017-08-25
MW075042	DozoGzzF	Culex tarsalis	USA	2017-10-26
MN233306	RT280	Culex tarsalis	Phoenix, AZ, USA	2017-06-27
MW075035	HRvuQr3H	Culex tarsalis	USA	2017-09-14
MW075020	PTIhijag	Culex tarsalis	USA	2017-08-10
MW075033	LUP2lQxG	Culex tarsalis	USA	2017-08-31
MW075023	XIqipGnc	Culex quinquefasciatus	USA	2017-08-23
MW075024	EMP6om9l	Culex quinquefasciatus	USA	2017-08-23
MW075037	6qGt3unV	Culex tarsalis	USA	2017-09-15
MW075039	Q67vi4NB	Culex tarsalis	USA	2017-09-22
MW075045	G937	Culex quinquefasciatus	USA	2018-10-02
MW075047	5475	Culex quinquefasciatus	USA	2018-07-26
MW075048	56	Culex quinquefasciatus	USA	2018-07-26
MW075068	Z587	Culex tarsalis	USA	2019-05-09
MW075074	X391	Culex tarsalis	USA	2019-05-15
MW075085	0E12	Culex tarsalis	USA	2019-07-10
MW075079	B468	Culex quinquefasciatus	USA	2019-05-16
MW075046	4255	Culex quinquefasciatus	USA	2018-07-25
MW075027	GfTYotIp	Culex quinquefasciatus	USA	2017-08-29
MN233312	IMPR165	Culex quinquefasciatus	Imperial Valley, CA, USA	2018-07-20
MN233313	IMPR570	Culex tarsalis	Imperial Valley, CA, USA	2017-09-11
MW074997	naHVJ2KH	Culex quinquefasciatus	USA	2015-06-12
MN233330	RT246	Culex quinquefasciatus	Phoenix, AZ, USA	2015-07-21
MW075013	fAhHi3Ji	Culex quinquefasciatus	USA	2015-07-21
MW075008	lhHIrQtH	Culex quinquefasciatus	USA	2015-06-24
MW074995	ijsN4KjT	Culex quinquefasciatus	USA	2015-05-07
MW075004	MGlBVL8d	Culex quinquefasciatus	USA	2015-06-19
MW075011	oC6yCbvY	Culex quinquefasciatus	USA	2015-07-10
MW075017	bUCbTx0k	Culex quinquefasciatus	USA	2015-07-23
KX258462	39	Culex quinquefasciatus	Maricopa Country, AZ, USA	2015-07-14
MW075001	Ld3BHJSA	Culex quinquefasciatus	USA	2015-06-17
MW075005	Nu6uTysq	Culex quinquefasciatus	USA	2015-06-19
MW075010	0hPYisVq	Culex quinquefasciatus	USA	2015-07-10
MW075015	Fa93JlIY	Culex quinquefasciatus	USA	2015-07-22
MW075016	SFFI0JoH	Culex quinquefasciatus	USA	2015-07-23
MW075006	dXKyQ7fA	Culex quinquefasciatus	USA	2015-06-23
MW075003	IGtvjeWM	Culex tarsalis	USA	2015-06-19
MW074996	udqWqPW5	Culex quinquefasciatus	USA	2015-06-12
KX258460	43	Culex tarsalis	USA	2015-07-14
MW075009	co5uNY8G	Culex quinquefasciatus	USA	2015-07-09
MW074998	rHqv8G4p	Culex tarsalis	USA	2015-06-16
MW075007	pJfJXuML	Culex quinquefasciatus	USA	2015-06-15
MN233307	BUCO327	Culex tarsalis	Butte County, CA, USA	2017-08-28
MN233322	TLRE179	Culex quinquefasciatus	Tulare County, CA, USA	2017-08-16
MN233309	DLNO229	Culex quinquefasciatus	Delano, CA, USA	2017-09-15
MN233310	FRWS650	Culex tarsalis	Fresno, CA, USA	2017-10-12
MN233323	TRLK660	Culex quinquefasciatus	Turlock, CA, USA	2017-08-03

GenBank ID	Strain	Host	Location	Collection date
MN233317	MADR393	Culex quinquefasciatus	Madera County, CA, USA	2017-09-29
MN233335	TLRE15	Culex quinquefasciatus	Tulare County, CA, USA	2018-06-20
MN233318	MERC342	Culex tarsalis	Merced County, CA, USA	2017-09-14
MN233325	WEST13	Culex tarsalis	Kern County, CA, USA	2016-07-20
MN233321	SUYA288	Culex tarsalis	Sutter/Yuba County, CA, USA	2017-07-31
MN233314	KERN245	Culex quinquefasciatus	Kern County, CA, USA	2018-07-05
MN233316	KERN351	Culex quinquefasciatus	Kern County, CA, USA	2017-06-21
KY825743	USA/CA/2016/human/UC-1	Human	USA	2016-09-09
MN233315	KERN345	Culex quinquefasciatus	Kern County, CA, USA	2016-07-15
KY825742	USA/CA/2016/mosquito-pool/UC-2	Culex pipiens	USA	2016
MW075000	ujdnAgDh	Culex quinquefasciatus	USA	2015-06-17
MW075012	qWYNXzzg	Culex quinquefasciatus	USA	2015-07-21
MW075019	xdVKzjJ2	Culex quinquefasciatus	USA	2015-10-20
MN233324	AR15-6004	Culex quinquefasciatus	El Paso, TX, USA	2015-07-21
MW075002	dgKktH00	Culex quinquefasciatus	USA	2015-06-19
MW075018	s8ZHrqTE	Culex quinquefasciatus	USA	2015-07-24
MN233319	NV16	Culex tarsalis	Clark County, NV, USA	2016-05-16
MN233311	ID17	Culex tarsalis	Gem County, ID, USA	2017-09-12
MN233320	OR17	Culex spp.	Malheur County, OR, USA	2017
MW075014	Eju6PeVN	Culex quinquefasciatus	USA	2015-07-22
KX965720	AZ14	Culex spp.	AZ, USA	2014
KT823415	RT 121B	Culex quinquefasciatus	USA	2015-07-07
MN233326	COAV2623	Culex tarsalis	Butte County, CA, USA	2015-08-25
MN233327	COAV2361	Culex tarsalis	Butte County, CA, USA	2015-08-04
MN233328	COAV2616	Culex tarsalis	Butte County, CA, USA	2015-08-25
KX258461	2281	Culex tarsalis	Coachella Valley, CA, USA	2015-07-28
MN233329	RT496	Culex quinquefasciatus	Phoenix, AZ, USA	2015-07-10
MN413675	SLEV/GIII/BuenosAires/Arg/001/2013	Human	Argentina	2013-03-25
FJ753286	CbaAr-4005	Culex quinquefasciatus	Argentina	2005
FJ753287	79V-2533	Culex spp.	Santa Fe, Argentina	1978

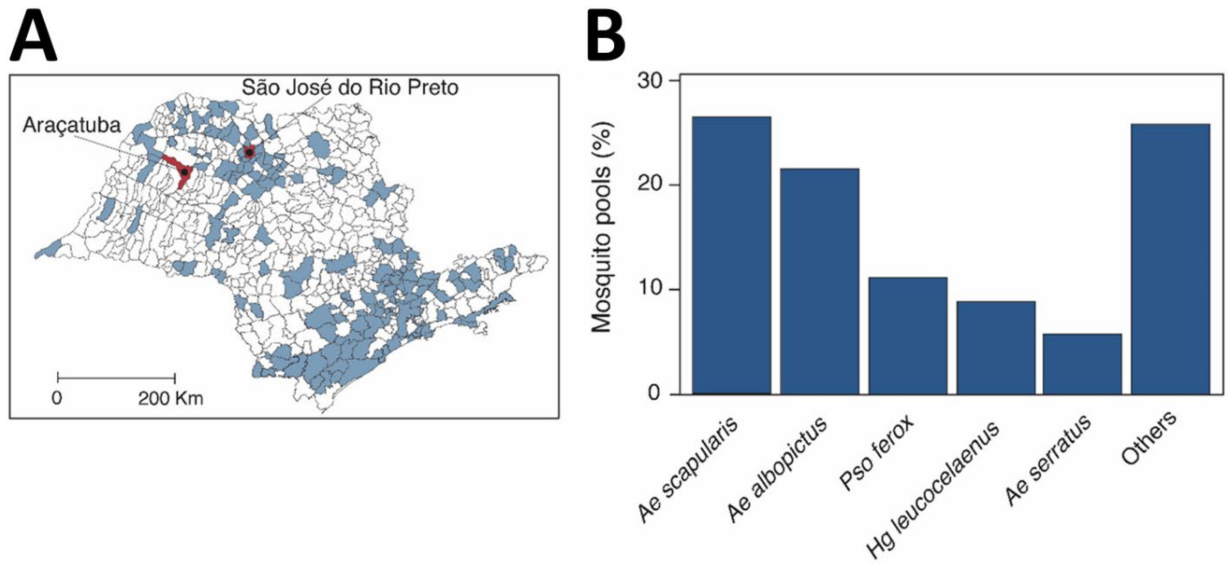

Appendix Figure. Mosquitoes captured in São Paulo State, Brazil. A) Map of mosquito collection sites positive for SLEV in São Paulo State, Brazil. B) Percentages of mosquito species pools identified morphologically in this study (total pools collected = 3,375). Detailed information on additional species and collection sites is provided in the Appendix. Km = kilometers; Ae = *Aedes*, Hg = *Haemagogus*, Pso, *Psorophora*.
